# The Effects of Food Environment on Obesity in Children: A Systematic Review

**DOI:** 10.3390/children10010098

**Published:** 2023-01-03

**Authors:** Johanna Key, Donna Burnett, Jeganathan Ramesh Babu, Thangiah Geetha

**Affiliations:** 1 Department of Nutritional Sciences, Auburn University, Auburn, AL 36849, USA; 2 Boshell Metabolic Diseases and Diabetes Program, Auburn University, Auburn, AL 36849, USA

**Keywords:** childhood obesity, body mass index, food desert, environment

## Abstract

Childhood obesity is an epidemic connected with poor eating. According to the United States Department of Agriculture’s Economic Research Service (USDA-ERS), food deserts are geographical locations in which residents have restricted or nonexistent access to healthful and quality food. Restricted access to healthy food is commonly associated with poor nutrition-related health outcomes, including obesity. This review aims to highlight the relationship between residing in a food desert or a similar environment on body mass index (BMI) in school-aged children in North America, predominantly in the Midwest region of the United States and Mexico. In this study, 17 articles were included from PubMed/Medline, Google Scholar, and Crossref. Most of these studies showed no association between the food environment and increased BMI. This discrepancy emphasizes the need for further research; the lack of access to healthful foods in food deserts is an issue that deserves additional attention.

## 1. Introduction

Childhood obesity is a growing concern that has begun to take shape over the past 45 years. According to the World Health Organization (WHO), in 1975, the prevalence of overweight and obese children was at 4% while fewer than 1% of children were obese. Data from 2016 illustrates a drastic increase to just over 18% or over 340 million children and adolescents being overweight or obese [1]. In United States, 19.7% of children and adolescents aged 2–19 years were classified as obese in accordance with the Centers for Disease Control and Prevention (CDC). Approximately 14.7 million children and adolescents are obese in the United States [2].

In the United States, childhood weight status is formally defined by the CDC growth charts as children the same age and sex having a BMI-for-age at or above the 85–95th percentile, which would categorize them as overweight, meaning they weigh more than 85–95% of kids their age in that reference population. Similarly, a child in the 95th percentile or greater is categorized as obese compared to other children in their reference population [3]. In contrast to the adult BMI scale, the BMI for children and teens is age and sex specific, which is why it is referred to as BMI-for-age. The increasing trend of children who are overweight or obese is concerning because childhood obesity is associated with many adverse health outcomes and diseases, such as obesity in adulthood, diabetes, heart disease, hypertension, dyslipidemia, and metabolic syndrome [4].

The United States Department of Agriculture’s Economic Research Service (USDA-ERS) defines food deserts as the geographical areas in which the residents have restricted or nonexistent access to healthful, affordable food options, such as fruits, vegetables, low-fat milk and dairy products, and whole grains, because of the absence of grocery stores and other entities within a convenient traveling distance [5]. To add perspective to that definition, a study tasked with assessing the extent to which residents of the United States are impacted by food deserts was presented in a report to Congress by the USDA-ERS [6]. This study showed that about 2.3 million people live greater than one mile from the nearest supermarket and do not have a car. Depending on where these people live, this could be a major issue. In urban locations, public transportation is more accessible. However, grocery stores in many cities may be distributed far or unevenly throughout the area, which may cause a travel burden even with public transportation. When living in a food desert, convenience stores, gas stations, and fast-food establishments often become the major source of nutrition due to availability. This results in the residents in these areas not receiving proper nutrition and facing financial hardship because, although the food is convenient, it is often not affordable.

Food deserts are commonly associated with food insecurity due to the limited availability of food options, but lately research has turned to investigating whether there is a connection between food deserts and obesity [7]. This is contrary to the usual association of undernutrition and food deserts. However, living in a food desert often increases a resident’s dependence on food outlets that often are not stocked with nutritious food that supports a healthy weight and lifestyle (i.e., snack foods, processed foods, and foods that are high in sodium and high fructose corn syrup). The increasing prevalence of childhood obesity warrants evaluation of the impact of these food deserts and their limited food selections on the children whose families are dependent on these outlets. The purpose of this systematic review is to assess the current published research to answer the question, “Does residing in a food desert, or an environment with food desert-like characteristics, lead to increased BMI in school-aged children?”

## 2. Methods

A systematic review was conducted, compiling research studies published from 2011 –2021 with one outlier published in 2009, due to the relevance of the study to the research and the limited number of more recent articles. Articles were selected that assessed the effect that residing in a food desert, or similar food environment, had on childhood weight status; therefore, the primary outcome measure necessary for each study to include as a dependent variable was Body Mass Index for age (BMI-for age). Other inclusion criteria included that the subjects were under the age of 13 years (eighth grade) and lived in a food desert or an environment that has characteristics that are similar enough to a food desert to be compared (i.e., limited access to grocery stores, increased availability of convenience stores and fast-food restaurants). Exclusion criteria included articles evaluating the effect of food deserts on specific disorders other than obesity, articles that included adults exclusively, food environment data not being included, and articles that did not include BMI. The databases used for article collection were Pubmed/Medline, Google Scholar, and Crossref. The key words used for the search included “childhood obesity and food deserts” and “neighborhood food environment and childhood obesity.” Due to the limited number of studies conducted on this topic, most of the studies found under this search criteria took place in North America, mostly in the Midwest and in Mexico.

## 3. Results

**Figure 1:**
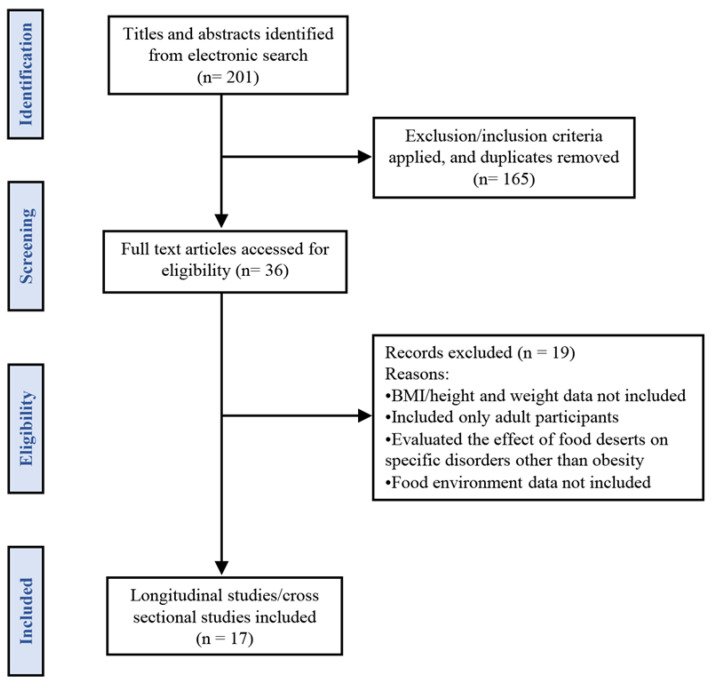
Flow chart for the article selection process.

We identified 201 articles in the databases and after manual screening 165 were removed based on the titles and abstracts. Therefore, 36 articles were included for further evaluation. Based on the inclusion and exclusion criteria 19 studies were excluded and only 17 articles included in the systematic review. Figure 1 shows the flow chart detailing the article selection process. A summary table of selected studies is provided in Table 1.

### 3.1. Study 1: Is there a Robust Relationship between Neighborhood Food Environment and Childhood Obesity in the USA [7]

Early Childhood Longitudinal Study Kindergarten Class was a multistage longitudinal study surveying a nationally representative sample of kindergarteners who were followed to eighth grade [8]. For this study the data were collected in the fifth and eighth grades. The residences of the students in eighth grade were geocoded to the census tracts. A total of 6260 (65%) of the students completed the data needed for this study and these students attended 1900 schools in 45 states in the United States and represented 2970 census tracts. The two outcomes evaluated in this study were BMI percentile in eighth grade and the change in BMI percentile from fifth grade to eighth grade. The food environment was measured using three measures: per-capita counts of a particular outlet type, indicators for specific outlet types of combinations, and food environment indices in a community. The results indicated that 36% of the sample were in the overweight category (BMI percentile > 85%) and 19% were obese (BMI percentile > 95%). More than two-thirds of the students (70%) reported living in a census tract that had one or more small food outlet or convenience store nearby and 36% of the participants had at least one supermarket. In addition, when evaluating the effect that living in a census tract with different combinations of food stores, the lowest BMI/change in BMI was associated with the students living in census tracts that had supermarkets only, as opposed to having convenience stores alone or a combination of supermarkets and convenience stores (*p* < 0.01). These coefficients were highly significant in a simple model but forfeited most of the significance when including other covariates or the longitudinal model. Due to this finding, an alternative hypothesis “more varieties of food outlets, regardless of type, predict higher BMI” was tested by utilizing an indicator for having grocery stores and convenience stores; supermarkets and convenience stores, supermarkets, and grocery stores; or all. The results indicated that in a cross-sectional analysis the coefficient was positive and significant (*p* < 0.005), indicating that when compared to a census tract where youth were exposed to at most one food outlet type, those exposed to more outlet types were associated with having a higher BMI [7].

### 3.2. Study 2: Food Deserts and Overweight School Children: Evidence from Pennsylvania [9]

Data were collected during the 1999–2000 school year from the Zip Code Business Patterns (ZCBP), Missouri Census Data Center, the National Center for Education Statistics (NCES), BMI data from the Pennsylvania Department of Health, and other sources. Demographics, households, and community environments were compared across all rural schools in Pennsylvania based on whether they met the criteria to be categorized as a food desert using geographic information systems. Weight status in the form of BMI-for age of 177,500 students within rural school districts was assessed, and the schools were categorized by whether they resided in a food desert. Food desert classification followed the Blanchard and Matthews approach developed in 2007 [10]. The results showed that people living in food-desert school districts had 12.76% of the population living below the poverty line compared to 10.69% of those in non-food desert school districts. The analysis of childhood weight status showed that about 16% of the schools and 14% of those participating lived in a food desert. Though the finding was not statistically significant, children who lived in non-food deserts exhibited healthier weights than those who lived in food deserts. In addition, the percentage of children who were overweight in food deserts was almost 2 percentage points greater than those who lived in non-food deserts, which was significant. The total percentage of students who were overweight in food deserts increased by 2.34% between 1999 and 2001, as opposed to the non-food desert districts which only saw a 1.08% increase. Researchers used a regression model to analyze the significance of students at risk for increased BMI based on whether they lived in a food desert. Each one-point increase in a school district’s population located in a food desert was associated with a 0.06% increase in students being at risk for or already overweight, which was a statistically significant finding (*p* < 0.01). Therefore, the results of this study demonstrated that food deserts are positively associated with an increased rate of overweight and obesity in school children [9].

### 3.3. Study 3: Food Deserts and Childhood Obesity [11]

Arkansas is one of the states in the United States with the highest poverty and obesity rates; therefore, it presents an interesting perspective. Due to the health status of the population in Arkansas, Act 1220 was passed, which is legislation aimed at reducing childhood obesity rates in the school system requiring that schools conduct BMI screening annually [12]. Data were collected from 2007 to 2009 on childhood obesity rates in school districts located in Arkansas from the Arkansas Center for Health Improvement (ACHI). The dataset consisted of 230 school districts, which included 25,299 students. Data were also collected on food store locations from Dun and Bradstreet (D&B) and the stores were screened based on whether they provided fresh fruits and vegetables. The USDA/ERS criteria for defining food deserts was utilized in this study. Three specifications were analyzed in the study: the baseline (Specification A) which included the percentage of the population with limited access to food stores, the free and reduced lunch participation rate, and the food desert indicator variable. Specification B only included the food desert binary variable, while Specification C centered around the percentage of the population with limited and free and reduced lunch participation. Despite the varying specifications, the researchers found no statistically significant associations between food desert status of an area, the degree of food store accessibility, or ratio of students qualifying for free or reduced lunch and obesity rates. This study attempted to be comparable to Study 2 by estimating the models using only rural districts as the sample with the three specifications detailed earlier. However, even when using only rural school districts there was no significant association linking school district obesity rates to any of the specifications. Overall, it was concluded that there was no statistical significance detected between school district obesity rates and food deserts located in Arkansas [11].

### 3.4. Study 4: Food Swamps and Food Deserts in Baltimore City, MD, USA: Associations with Dietary Behaviors among Urban Adolescent Girls [13]

This study used a cross-sectional design to assess the effect that the food environment surrounding the home relates to food consumption in low-income, urban, African American girls in early adolescence. Low-income neighborhoods have less access to supermarkets that sell healthy food but have more retailers that sell less healthy and energy-dense foods are labeled as ‘food swamps’ as the availability of healthy food is replaced by less healthy foods [14,15]. The hypothesis of this study was that living in a food desert is associated with decreased fruit and vegetable intake, and that living in a food swamp is associated with higher snack and dessert consumption. Data were collected from a multilevel randomized controlled trial focused on health promotion and obesity prevention in sixth and seventh grade girls. The outcomes measured included BMI-for-age used to assess weight status, and dietary intake which was evaluated by the students’ responses to the Youth/Adolescent Food Frequency Questionnaire [16]. In addition, the students’ food environment was mapped via geocoding using the ArcGIS geographic information system (GIS) version 10.0. Over half of the participants (52.4%) were either overweight or obese. Over one-quarter (26.5%) of the girls were living in a food desert, while 35.2% of the girls were living in a food swamp. The results of the food consumption analysis showed that girls living in a food desert or food swamp consumed more snacks and desserts than girls who lived in neighborhoods that were not considered swamps or deserts (β = 0.13, *p* = 0.029). Furthermore, girls with a lower BMI-for-age z-score were found to consume more snacks and desserts than the girls with higher BMI-for-age z-scores. These results suggest that the girls with lower BMI z-scores lived in food deserts and swamps. In addition, proximity to one or more convenience store was associated with a statistically significant increase in consumption in snacks and desserts (β = 0.16, *p* = 0.003), following other trends that childhood BMI may be impacted by proximity to unhealthy food options rather than food desert status alone. The results of the study demonstrated that adolescent girls living in food swamps and deserts consume more snacks and desserts than those who do not live in food swamps/deserts. However, in this study, food desert status was not associated with increased BMI z-scores [13].

### 3.5. Study 5: The Effects of Food Deserts on the Weight Status of South Dakota Children [17]

The South Dakota Department of Health collected height and weight data from a total of 1408 students from six schools for the 2010–2011 school year. These measurements were utilized to calculate weight status in terms of BMI-for-age. The students’ weights were categorized as underweight, healthy weight, overweight, or obese. The residences of the students were classified as border-food desert areas, non-food desert areas, and food desert areas and the quality of the food providers in the area was assessed. When assessing the percentage of students who were classified as obese in the three specific zones detailed above, the results demonstrated that the border-food desert areas had the lowest total percentage of students who fell in the obese category. Meanwhile, the non-food desert areas had the highest percentage of students who classified as obese and those who lived in a food desert area fell between those two categories. Therefore, there was no statistical significance between living in a food desert and childhood obesity as measured by BMI [17].

### 3.6. Study 6: The Effect of Food Deserts on Body Mass Index of Elementary School Children [18]

This study utilizes the USDA/ERS classification for the framework of the study. BMI z-scores were collected from the Arkansas childhood BMI data set from the 2003–2004 school year to the 2009–2010 school year, for a total of 7 years. Gender, age, race, ethnicity, and school meal status (qualifying for free or reduced school lunches) was also determined. Similar to other studies, Dun and Bradstreet (D&B) was used to obtain food store location data. The results demonstrated that there was no statistical difference from zero when evaluating the low-food access measure across the samples. These results indicated that living nearby supermarkets alone was not associated with increased BMI across the sample. However, in low-income communities, the absence of supermarkets was associated with weight gain, likely because of other factors also part of low-income communities. In addition, there was no statistically significant association between the distance to the nearest supermarket and BMI. When evaluating food deserts’ effects on BMIs of children living in urban versus rural areas, the results demonstrated that food deserts were more strongly associated with high BMI in children living in urban areas than in rural areas (*p* < 0.01). Although the results suggest that living in a food desert may cause weight gain, the evidence not strong enough to be considered causal and may be due to other confounding factors within the community.

### 3.7. Study 7: The Toxic Food Environment around Elementary Schools and Childhood Obesity in Mexican Cities [19]

Cross sectional studies took place in two Mexican cities in which prevalence of overweight and obesity in school children is high (Cuernavaca, Morelos = 31.8%; Guadalajara, Jalisco = 37.0%) according to the 2006 National Health and Nutrition Survey. A total of 725 school children completed the study from 60 schools. Fourth and fifth grade students were randomly selected from classes within the schools with an average of four students participating per class. BMI z-scores-for-age and sex were used to classify overweight and obesity in children, and the mobile food venders were classified in three different categories: unhealthy foods, healthy food, and mixed food venders. Food stores, such as grocery and convenience stores, were also included in the analysis. This study was not evaluating food environment in terms of “food deserts” per se; however, due to the limited availability of articles on the topic and the food environment having elements that are comparable to food deserts it was included. Of the 725 students studied, 24.8% were overweight and 20.7% were obese. The results showed that there were significantly more food venders near public schools as opposed to private schools (*p* < 0.05). Most of these venders (85%) were categorized as serving unhealthy foods. Using a linear regression model, the results showed a significant positive statistical association between number of food vendors serving unhealthy food around the school and BMI of the school children (*p* < 0.05). However, interactions between the local food environment-related variables and student BMI at the school level were not statistically significant. This may suggest that there may be other explanatory variables not included in the study other than the school’s category, which the researchers used as a proxy for socioeconomic status.

### 3.8. Study 8: The Role of Local Food Availability in Explaining Obesity Risk among School-Aged Children [20]

This study used ECLS-K data, similar to the Study 1. The study sample was drawn from the spring enrollment from U.S. school children in kindergarten, first, third and fifth grades, which included 11,400 children. The primary outcome was monitoring BMI-for-age over time. Neighborhoods were classified using the 2000 Census tract-level information. In addition, information from Dun and Bradstreet’s NETS dataset was also included for determination of local food environments. According to the results, on average, the school children experienced increasing BMI rankings over the course of their time in elementary school. The average increase was 4.76 points but there was a disproportionate increase for poor (7.01), black (7.85), and Hispanic (5.88) children compared to non-poor (4.43) and white (3.85) counterparts. The minority groups seeing disproportionate weight gain were gaining weight at a rate that outpaced height growth. The minority populations resided in locations in which there was an abundance of fast-food restaurants and convenience stores. However, these minority groups also had significantly greater access to food establishments that were not linked to obesogenic lifestyles. There were no significant differences by race or poverty status within the neighborhoods, but the corner stores in the impoverished and minority communities had significantly higher per capita prevalence, with convenience stores more widespread in poor areas. When evaluating changes in BMI percentile based on food outlet exposure, there was no independent relationship to child weight gain. The findings of this study demonstrated that the presence of supermarkets was not limited in low-income/minority areas, which shifts the nutritional focus from not having enough to having too much access to foods that are not in line with a healthy diet.

### 3.9. Study 9: Neighborhood Convenience Stores and Childhood Obesity: A Panel Data Instrumental Variable Approach [21]

This study took place in Arkansas with BMI data collected from the Arkansas childhood BMI database for 7 academic years (2003–2004 to 2009–2010). Gender, age in months, race, and school meal status were also recorded. Food store location information was purchased from Dun and Bradstreet(D&B). The residence of the students was geocoded to determine how many convenience stores were in proximity of each resident. The models of the study included a control for fast-food restaurants which play a role in the overall food environment. Follow-up analyses examining food residences separately were also included in the study though the primary outcome measure was convenience store impact on BMI. USDA/ERS’s Food Desert Atlas was used to classify food desert status. The results suggested that children living near convenience stores had a BMI z-score that was 0.144 standard deviation higher than those without access, which was statistically significant (*p* < 0.001). When evaluating the impact of distance, a one-mile increase in distance between a child’s residence and a convenience store reduced BMI z-score by 0.012 standard deviation (*p* < 0.001). In terms of children living in food deserts and economic status, there was no evidence that convenience stores disproportionately affected children in households with lower economic status compared to children in households with better economic status. Therefore, though it was found that proximity to convenience stores was associated with slight increases BMI in schoolchildren, there was no evidence supporting the idea that children from low-income families or children living is food deserts experience disproportionately large impacts.

### 3.10. Study 10: School and Residential Neighborhood Food Environment and Diet among California Youth [22]

This study took place in California where data were selected from the 2005 and 2007 waves of the California Health Interview Survey (CHIS). The primary outcome was self-reported consumption of fruits, vegetables, 100% juice, milk, soda, high-sugar food, and fast food. BMI percentile was the second outcome measure used in the study. Adolescents and parents (for the children) were randomly selected to be interviewed about their dietary intake. Parents reported height and weight data used to calculate BMI for the children in the study. The neighborhood food environment was assessed using ArcMap and food outlet data collected from InfoUSA’s 2006 release. When evaluating proximity of food stores to the residence, it was shown that 70% of families with children had no supermarkets within 0.5 miles from home. However, there were no significant findings for the relationship between neighborhood food environment and dietary intake or BMI in California children or adolescents in the study. Regardless, covariate effect size estimates supported a trend across the models showing that gender, age, and parents’ BMI are stable predictors of their children’s BMI percentile. The study concluded no robust relationship between food environment, consumption, or BMI percentile of children or adolescents was present.

### 3.11. Study 11: Food Environment and Childhood Obesity: The Effect of Dollar Store [23]

This study took place in Arkansas by the same group of researchers as Studies 3, 6 and 9. BMI data were collected by the Arkansas Center for Health Improvement via the BMI screening program for public school children mentioned above. In this study, the effect that access to dollar stores (DS) has on childhood BMI status was assessed. Children were exposed to a DS if there was a DS within one mile of their residence or a 10-mile radius for children in rural areas, similar to the criteria used by the USDA-ERS to define food deserts. There were three different age cohorts of children, each observed for a total of 5 years: 2004–2008, 2005–2009, and 2006–2010 with the starting year of each cohort being the year the children started kindergarten. The results indicated that there was no evidence that supported the idea that presence of a DS near a child’s residence was associated with increased BMI. Instead, there was an increase in BMI when a DS leaves a child’s neighborhood. In conclusion, there was no significant evidence found supporting the idea that access to a DS had a profound effect on increased childhood BMI status. In fact, the inverse seemed to be more probable.

### 3.12. Study 12: From Food Desert to Food Oasis: The Potential Influence of Food Retailers on Childhood Obesity Rates [24]

The purpose of this study was to examine the relationship between food environments and obesity rates in preschool-aged children living in low-income communities. The main effects included (a) a change in the number of grocery stores and supermarkets, (b) a change in the number of supercenters and club stores, and (c) the number of convenience stores present per capita. A total of 2713 U.S. counties were represented in the analysis. The BMI data were collected using the 2009–2011 Pediatric Nutrition Surveillance System (PedNNS) data for children from ages 2–4. Population data were collected from the U.S. Census Bureau, County Intercensal Estimates, and information about food environment was drawn from the U.S. Census Bureau, County Business Patterns. It was predicted that the percent change in the number of grocery stores/supermarkets and supercenters/club stores was related to the obesity rate in low-income, preschool aged children in the U.S. The results supported this prediction by demonstrating that the coefficient for the change in number of grocery stores/supermarket was negative (−0.054) and significant (*p* < 0.01) meaning that as the change in the number of grocery stores/supermarkets increased (decreased), the rates of obesity within the communities decreased (increased). In addition, the results showed that there was a significant, positive relationship between the number of convenience stores in a food environment and childhood obesity rate among low-income children (*p* < 0.001). 

### 3.13. Study 13: The Relationship of Lack of Access to Affordable and Healthy Foods and Obesity Rates in Tennessee Adults and Children [25]

The goal of this study was to explore how obesity rates correspond with the percentage of a population living in food deserts. Location data were drawn from the archival data from the Food Desert Locator of the USDA-ERS. Population data were collected using the U.S. Census Bureau 2010 release and childhood weight status information was collected from the Tennessee Coordinated School Health Childhood Obesity Rates by County Data from the 2008–2009 release. It was noted that percentage of households that had at least a bachelor’s degree and households without a vehicle were significant predictors of both childhood and adult obesity. When evaluating the relationship between living in a food desert and obesity rates, it was found that there were no significant associations between the number of children/adults living in a food desert and obesity rates in that location. However, childhood obesity was significantly correlated with adult obesity (*p* < 0.05). When evaluating the relationship between type of food deserts and obesity rates within the area, the only variable of significance was the percentage of children in urban food deserts, indicating that as the percentage of children living in an urban food desert increased, childhood obesity rates decreased (*p* < 0.05). The study concluded that the type of food desert environment and resources available within the community may be more influential on obesity rates than simply residing in a food desert.

### 3.14. Study 14: Proximity to Supermarkets Associated with Higher Body Mass Index among Overweight and Obese Preschool Aged Children [26]

The objective of this study was to analyze the associations between proximity to food establishments and BMI among preschool-aged children. A total of 438 children from ages 2–6.9 years with BMI ≥ 85th percentile was included in the study. These children had participated in a randomized control trial in Massachusetts from 2006–2009. GIS data were used to determine the children’s proximity to the following food establishments: (1) convenience stores, (2) bakeries, coffee shops, candy stores, (3) full-service restaurants, (4) large supermarkets, (5) small supermarkets, and (6) fast-food restaurants. The main outcome variable was BMI. The results indicated that 35% of the participants lived less than 1 mile away from a large supermarket, 42% lived between 1 and 2 miles away from a supermarket, and 22% lived more than 2 miles away from a supermarket. Children who were found to live more than 2 miles away from a large supermarket had a BMI that was 1.06 kg/m^2^ greater than those who lived within 1 mile of a supermarket, which was a significant finding (*p* < 0.05). With this finding, it was concluded that living closer to a large supermarket was associated with a decreased BMI in preschool-age children who were overweight or obese.

### 3.15. Study 15: Does Proximity to Fast Food Cause Childhood Obesity? Evidence from Public Housing [27]

The purpose of this study was to examine whether there is a causal link between proximity to fast food restaurants and incidence of childhood obesity amongst low-income households in New York City (NYC). Data on students who reside in public housing were collected because the public housing system in NYC provided a simple, yet effective way to randomize individual proximity to fast food restaurants in a development. Weight and height data for the students were collected using the FitnessGram, which is a program that collects weight and height data for students within the school system yearly. Food environment information was collected from the NYC Department of Health and Mental Hygiene. The results revealed that childhood obesity increased as a child’s residence grew closer to a fast-food restaurant, with more significance shown in the younger children (*p* < 0.01). In fact, every additional 0.1-mile separation between a child’s residence and the nearest fast-food restaurant was associated with a decrease in the probability of children being obese by approximately 0.6 percentage points. The study concluded that proximity to fast food restaurants positively correlates with childhood BMI status. Therefore, as proximity increased, BMI increased as well.

### 3.16. Study 16: Obesogenicity and Assessing its Impact on Child Obesity: A Cross-Sectional Ecological Study for England Neighbourhoods [28]

This cross-sectional ecological study was primarily focused on developing a methodology for quantifying obesogenic neighborhoods in England. Through quantification of these neighborhoods, the association between characteristics of an obesogenic neighborhood (poor access to healthy food and recreation) and childhood obesity was detailed. In this study, food deserts were defined in relation to the varying spatial access to healthy food (e.g., supermarkets) versus less healthy options (such as fast-food restaurants). Data on children ages 10–11 years old living in English neighborhoods were collected utilizing the National Child Measurement Programme (NCMP) [29]. Measures of childhood weight status, and other factors, were collected for all children in primary schools managed by the state which equates to 94% of primary school children in England. The framework is provided by Middle Level Super Output Areas (MSOAs) which collected data on 6791 neighborhoods providing a complete representation of primary students in England [30]. The results of the parameter measuring the average childhood obesity rates in obesogenic neighborhoods compared to that of nonobesogenic environments reflected 25.5% and 15.7%, respectively, demonstrating 9.8% difference. They also evaluated obesogenicity in rural areas versus suburban areas; it was found that urban locations were more likely to be obesogenic. The study concluded that when living in an obesogenic area [combined with income deprivation], there is a secondary significant effect on childhood obesity.

### 3.17. Study 17: Exploring the Associations between Neighbourhood Food Environment, Household Food Insecurity, and Child Weight-Related Outcomes in Socio-Economically and Radically Ethnically Diverse Families [31]

This cross-sectional, observational study took place using a racially/ethnically diverse sample of U.S.-born and immigrant/refuge families to examine the connection between neighborhood food environments (NFE), household food insecurity (HFI), and weight outcomes in children. This study included six ethnic/racial groups including African American, Latino, Hmong, Native American, Somali/Ethiopian, and White. A total of 1296 families with children ages 5–9 years old were selected using data collected by the Family Matters study from 367 census tracts from the Minneapolis/St. Paul, MN metropolitan area [32]. In this study, food environment was defined by the distribution of food outlets around a resident’s home, with distance between one’s home and food retail/service outlets and the types of food establishments present in the area [supermarkets, fast food outlets, and convenience stores] also included. The results of this study showed that neighborhoods with limited access to supermarkets with fresh fruits and vegetables were associated with lower food security at home (*p* < 0.01). In relation to childhood BMI status, it was found that there was a significant association between living in a food desert and children with higher BMI percentiles if the household was food insecure (*p* < 0.05). This study concludes that both NFE and HFI can have a negative impact on childhood weight outcomes, and both should be considered when crafting interventions.

### 3.18. Results Summary—Food Environment, Proximity, and Intake

In summary of the results of the 17 studies presented in this review, BMI percentile in children was assessed based on the general food environment [food deserts, food swamps, and non-food deserts made up of supermarkets, grocery stores, fast food restaurants, convenience stores, and food venders]. The results based strictly on food environment were varied with no overwhelming significant findings overall. Some of the studies also detailed proximity to specific food sources like DS, convenience stores, grocery stores, or fast-food restaurants. When proximity was included as a factor, results showed that BMI percentile was increased in children living further from supermarkets or closer to fast food, convenience stores, or unhealthy food vendors. A couple of studies took a closer look at intake which provided a clearer picture of what type of food choices take place in different food environments, but they did not find much evidence that these food choices had a significant effect on BMI status of the children living there.

**Table 1:** A summary of the selected studies and important parameters.

Study, Year	Number of Participants	Data Periods	Data Sources	Food Environment	Outcome Measure(s)	Research Question Answer	Additional Support
Study 1: 2012 [7]	6260	3 years	ECLS- K, Info USA, NAICS	food desert (grocery store/convenience/fast food exposure)	BMI	No	
Study 2: 2009 [9]	177,500	1 year	ZCBP, Missouri Census Data Center, NCES, PA- DOH	food deserts school district/nonfood deserts school district	BMI	Yes	
Study 3: 2012 [11]	25,299	2 years	D&B, Arkansas Childhood BMI Database	food deserts (limited food stores, free/reduced school lunch/food desert	BMI	No	Arkansas’s Act 1220
Study 4: 2016 [13]	634 African American girls	N/A	Youth/Adolescent FFQ (YAQ), ArcGIS	food swamp/food desert/neither	BMI/dietary intake	Maybe	Sample size representing minority group
Study 5: 2016 [17]	1408	1 year	South Dakota DOH	border-food deserts/non-food deserts/food desert areas	BMI	No	
Study 6: 2015 [18]	110,384	7 years	Arkansas Childhood BMI Database, D&B	supermarket exposure	BMI	Maybe	Revisited topic with an adapted study design
Study 7: 2016 [19]	725	1 year	Ministry of Education in Mexico	unhealthy/healthy vendors exposure	BMI	Maybe	
Study 8: 2012 [20]	11,400	1 year	ECLS-K, D&B	food desert (grocery store/convenience/fast food exposure)	BMI	No	
Study 9: 2019 [21]	855,138	7 years	Arkansas Childhood BMI Database, D&B	convenience store exposure	BMI	Maybe	Revisited topic with an adapted study design
Study 10: 2012 [22]	8226	N/A	California Health Interview Survey	grocery stores/fast food	BMI/dietary intake	No	
Study 11: 2015 [23]	99,644	5 years	Arkansas Center for Health Improvement Arkansas Childhood BMI Database, D&B	Dollar stores	BMI	No	
Study 12: 2016 [24]	N/A	2 years	Pediatric Nutrition Surveillance System (PedNSS), SNAP, County Business Patterns	food desert (supermarket/ grocery store/convenience store exposure)	BMI	Yes	
Study 13: 2017 [25]	3939	1 year	US Census, Food Desert Locator, Tennessee Coordinated School Health Childhood Obesity Rates	Food deserts (urban vs. rural)	BMI	Maybe	
Study 14: 2013 [26]	438	3 years	BMI Data from RCT in Massachusetts, GIS,	Food environment (convenience stores, full-service restaurants, large supermarkets, etc.)	BMI	Yes	
Study 15: 2020 [27]	486,048	N/A	Public housing system in NYC, FitnessGram, Department of Health and Mental Hygiene	Fast food exposure	BMI	Yes	
Study 16: 2022 [28]	N/A 6791 neighborhoods	N/A	Middle Level Super Output Areas (MSOAs), National Child Measurement Programme (NCMP)	Supermarket vs. fast food exposure	BMI	Yes (secondary)	
Study 17 2022 [31]	1296 families with children	3 years	Minneapolis/St. Paul, MN metropolitan area census tract, Family Matters study	Food environment (supermarket, fast food, convenience stores, access tom fruits and vegetables)	BMI	Yes	Racially and ethnically diverse sample

## 4. Discussion

The results of this review of the literature on childhood obesity and food environment illustrate mixed conclusions. Six out of the seventeen studies included found a robust association between food environment [access to grocery store, fast food, and convenience stores] and elevated BMI in children. Five studies could not come to a definite “yes or no” significance between the relationship in question. The last six studies reported no association between the two. Some studies found that children who lived in a food desert or in an area with limited access to healthful food exhibited higher BMI, but these studies also included other parameters such as racial and ethnic diversity/ethnicity, income status, and access to recreation [26,31]. When including studies that concluded in a “maybe”, in more than half of the studies that found no statistically significant associations, the children with the higher BMI were the children who had more access to food as opposed to those living in a food desert. In addition, many of the studies found that the access to supermarkets did not have a “protective effect”.

There were studies that yielded results, or trends, that were significant; however, the impact that the food environment had on the subjects was small, suggesting it played a minor role in the overall childhood obesity issue. Many of the trends were presented in the studies that took place in Arkansas. Arkansas is known to have some of the highest poverty and obesity rates in the United States. Perhaps the studies in Arkansas yielded different results because of the exaggerated nature of the issues [11,16,19,21]. The other study located in Pennsylvania used an abstract view of food deserts (rural food deserts) which was hard to operationalize and compare to the rest of the current research [9]. However, this does not discredit the importance of these positive findings. These findings provide evidence that spatial inequality of resources plays a role in health outcomes and disparities. In addition, another study that found positive significant results only found them when evaluating the effects that specific food establishments, such as fast-food restaurants alone had on childhood weight status which may indicate another issue dealing with that specific establishment by itself rather than the food desert status of an area [19].

The limitations of this study include that many of the studies used collected geographic information on the food environments from InfoUSA or Dun and Bradstreet, which are commercial vendors that may not be as accurate as they are marketed to be. Therefore, the neighborhood food environments may not be accurate. In addition, due to the limited number of studies performed on this specific topic, not all the studies analyzed environments that were identified as “food deserts”. However, the focus of these studies was considered to be close enough to be used in this analysis. In addition, many of the studies available did not have diverse samples that included a variation of different racial and ethnic groups, failing to account for the possibility that systematic racism may also play a part in accessibility to healthful food options.

The strengths of this study were that many of the variables within the studies were comparable across most of the studies. For example, most of the studies utilized the USDA/ERS definition of food deserts. The limitation mentioned that many of the studies used InfoUSA or Dun and Bradstreet may also be considered a strength in terms of comparable data collection. In addition, all the studies had large to very large sample sizes. Another strength of the present study was that Studies 3, 6, 9 and 11 were performed by the same group of researchers [11,16,19,21]. Study 3 took place in 2013 and used a panel data set with classical and spatial panel data models in which there were no significant findings related to food deserts and increased BMI [11]. The researchers repeated the study in 2015 and added fixed-effect regression models and with that, they found results that were not robust enough to be considered causal, but that followed a trend that supported the idea that living in a food desert may be associated with weight gain. Additionally, in 2015, this group of researchers published a study evaluating the effect that DS specifically have on childhood weight status [16]. However, these results showed no association between the presence of DS and increased BMI. In fact, the absence of DS was more strongly associated lower BMI in children. The final study was published in 2019, and in this study the researchers only evaluated the effect that convenience stores had on childhood BMI as opposed to food deserts as a whole and the results, though miniscule, also supported the idea that convenience store exposure was associated with increased BMI in school children [25]. Inclusion of these three studies emphasizes the importance of revisiting topics with improved methodology.

## 5. Conclusions

Overall, residing in a food desert, or a place with food desert-like characteristics (abundance of convenience stores/increased rates of poverty) was not significantly or robustly associated with increased BMI in children. This conclusion is based on a study’s ability to answer the research question presented in this review. Although there were cases in which the students’ BMIs were shown to increase within a food desert, there was an equal amount of evidence pointing to the fact that many other factors may have attributed to childhood obesity. There seemed to be stronger evidence that increased BMI may be due to having more access to food as opposed to limited options. The results are surprising, but it brings up the idea that it is more than just the food environment within a food desert that has an obesogenic effect on the population. Other aspects of the community must be evaluated in a broader approach to the issue of obesity in children. Although the results did not overwhelmingly indicate that food deserts are a major cause of childhood obesity, the issues within food deserts cannot simply be ignored. Food deserts still represent the inequity within our society where everyone who should have access to healthful, affordable food does not. Perhaps indicators other than BMI should be used to evaluate the nutritional status of children such as evaluation of nutritional intake in comparison to nutritional requirements, economic standing of families, and the presence of racial disparities and ethnic inequalities. The relevant nutritional issue may have to do more with micronutrient intake than caloric intake. As well, physical activity needs to be evaluated and controlled in BMI studies.

## Data Availability

Not applicable.
